# Supplementary material for: An RNA‐binding atypical tropomyosin recruits kinesin‐1 dynamically to oskar mRNPs
Source: EMBO J. 2016 Dec 27;36(3):319–33. doi: 10.15252/embj.201696038 (PMC5286366; doi:10.15252/embj.201696038)
Supplement: Supplementary file 1 — Appendix [file EMBJ-36-319-s001.pdf]

An RNA-binding atypical tropomyosin recruits kinesin-1 dynamically to *oskar* mRNPs

Gaspar et al.

Appendix

Table of contents

Appendix Supplementary Materials pages 2-3

Appendix Tables pages 4-5

Appendix Figure Legends pages 6-7

Appendix Figures pages 8-9

Appendix Supplementary Materials

Transgenic constructs and new alleles:

UASp-EmGFP-Tm1-I and *UASp-mKate2-Tm1-I* were created by fusing the EmeraldGFP and the mKate2coding sequences in frame with the 5' of the Tm1-I coding sequence (cds), respectively, followed by the 3' UTR amplified from an ovarian cDNA. These construct were completed with the 5' UTR of Tm1-I and inserted into pUASp2 transgenesis vectors (Rorth, 1998).

UASp-Khc_{401}*-MCP* was cloned by ligating the sequence encoding the N terminal-most 401 aminoacids of Khc - sufficient for processive movement (Telley et al., 2009) – with the cds of MS2-coat protein (MCP) in frame in a pUASp2 transgenesis vector.

The *oskar* RNA null allele, *oskar*^{attP,3P3GFP} (*oskar*^{0}) was created by homologous recombination mediated knock-in. A double-stranded break in the *oskar* promoter 26 nucleotides upstream of the transcriptional start site was induced by CRISPR/Cas9, by co-injecting a Cas9 expressing vector, a U6 promoter driven guide RNA (5'GAATGGAGAAGTGGACCGCGT**NGG**) and a template plasmid carrying a minimal attB-loxP-GMR-3P3-EGFP-tubulin3'UTR-loxP cassette flanked by two ~1000 bp long homology arms (Sebo et al., 2014). Female flies homozygous for *oskar*^{attP,3P3GFP} are sterile, oogenesis is terminated prematurely at around stage 6, and oocytes express the same karyosome defects as previously reported by Jenny et al. (Jenny et al., 2006). The *oskar* promoter drives EGFP expression from the *3P3-EGFP* marker gene in the female germline which may interfere with the read-out of another GFP tagged transgene. Cre mediated removal of the *3P3-EGFP* marker gene restores female fertility.

The genomic Tm1 locus was tagged with the *mCherry* cds (*Tm1*^{mCherry}) using a similar strategy as described for *oskar*^{attP,3P3GFP}, using the guide RNA cds (5'GGAGTCATCGTGCTCCATGT**NGG**) and a loxP-GMR-3P3-EGFP-tubulin3'UTR-loxP-mCherry cassette flanked by two ~ 600 bp long homology arms targeting the 5'UTR/ATG boundary to insert the *mCherry* sequence in frame with Tm1-I. Expression of mCherry-Tm1 was achieved by Cre mediated stable removal of the *3P3-GFP* marker gene from the locus. Flies homozygous for *Tm1*^{mCherry} are viable, however have reduced life span and are flightless, probably due to internally tagged and thus compromised Tm1-E and Tm1-H isoforms. Both homozygous males and females are fertile.

The genomic Khc locus was tagged with the *mKate2* cds (*Khc*^{mKate2}) using a similar strategy as described above. The genome was targeted using a guide RNA cds (5 GATTGGATCTACGAGTTGAC**NGG**) and an mKate2 cassette flanked by two ~ 700 bp long homology arms targeting the STOP/3'UTR boundary to insert the *mKate2* sequence in-frame with Khc. F1 generation embryos were screened for the mKate2 fluorescence to identify individuals with modified genome. Flies homozygous for *Khc*^{mKate2} are viable and fertile.

Appendix Tables

genotype	No. of oocytes	No. of runs	Fraction of motile RNPs (%)[a]	Displacement (µm) [b]	Duration (s) [b]	Velocity (µm/s)[a]	Net velocity vector (nm/s)[c]
wild-type*	13	681	31.95±2.90	1.054±.682	2.179±1.558	.55± .01	51.2±107.8
$Tm1^{eg9}$/+	10	157	14.25±2.84	1.225± .999	2.910± 2.586	.49± .02	-58.4±95.28
$Tm1^{eg9}$/ $Tm1^{eg1}$	15	22	1.89±0.47	0.864± .707	1.816± 1.820	.62± .09	0.3± 41.0

Table S1, related to Fig 1: Motility statistics of *oskar* RNPs in *$Tm1^{gs}$* mutant oocytes *in vivo*

[a] – mean±SEM

[b] – mean±SD

[c] – mean±SD, the resulting net velocity vector after vectorial averaging of the velocities of *oskar* RNP runs in individual oocytes of a given genotype (Zimyanin et al., 2008). None of the net velocity vectors are significantly different from zero (2-tailed Wilcoxon sum ranks test, p>0.05)

* - data taken from (Gaspar et al., 2014)

name	sequence (5'->3')
osk3'UTR_1	ATCGCGCAAATGCTTCACU
osk3'UTR_2	TTAAGGGCAAGTGGCAGGU
osk3'UTR_15	ACGTGATCACCATCAATACAU
osk3'UTR_16	AGCTGTAAATTACGCCAGAAU
osk3'UTR_17	TGCTACAAACAAGCGCTTAGU
osk3'UTR_18	TCTGCAGCAGAGTGTAAGCAU
osk3'UTR_19	AATTTGCTTGAGCACATCAAU
osk3'UTR_20	GTTGATTTTGTGCAAGCGAAU
osk3'UTR_21	TTCCAAGTAAAGCAGTGCAU
osk3'UTR_22	TTACGGCCAAAATGCAGCAU
osk3'UTR_23	TGTATACGTACCACGCCCACU
osk3'UTR_24	TGATACAGGAGCATGCCGAAU
osk3'UTR_25	GCGGAAAAGTTTGAAGAGAAGU
osk3'UTR_26	CTGCTTGCGCTTATTTTGCAU
osk3'UTR_27	CGAATTCCGTAAAAGCCGAU

Table S2: Sequence of smFISH probes targeting the *oskar* 3' UTR.

Underlined U at the 3' end of the probes indicates the TdT incorporated Atto565-ddUTP.

Appendix Figure Legends

Appendix Figure S1, related to Figs 1, 2, 3, 4 and 5:

(A) Current gene model of the *DmTopomyosin1* locus encoding muscular (E, S, N and L), cytoskeletal (A, R and Q) and non-classified isoforms (H, C and I). Blue triangle indicates the P-element insertion of the *Tm1*^{gs1} allele, the founder of *Tm1*^{eg1} and *Tm1*^{eg9}. Red triangle indicates the insertion of the *mCherry* coding sequence, resulting in *Tm1*^{mCherry}.

(B) Semi-quantitative RT-PCR of different transcript groups (as indicated in a) of *DmTm1* in wild-type (wt), *Tm1*^{eg1}/*Tm1*^{eg1} *(eg1)* and *Tm1*^{eg9}/*Tm1*^{eg9} *(eg9)* ovarian extracts.

(C) Western blot of ovarian lysates using a pan-Tm1 antibody. The main signal at 34 kDa represents Tm1-A and Tm1-L isoforms. The band most likely representing Tm1-I is indicated with an arrowhead. This band is almost completely absent in *Tm1*^{eg9}/*Tm1*^{eg9} (lane 2) and *Tm1*^{mCherry}/*Tm1*^{mCherry} (lane 4) lysates. mCherry-Tm1 (lane 4) and the over-expressed EmGFP-Tm1-I (lanes 5-6) are indicated with a white arrow.

(D) Western blots of Khc, EmGFP-Tm1-I and Y14 in *oskar* RNA null, *y14* RNAi and wild-type egg-chambers. (E) Western blot of Khc, Vasa and α-Tubulin in *Khc* RNAi egg-chambers. Expression of Khc, Khc-EGFP, Oskar (F) and Khc-mKate2 (G) in wild-type and *Tm1*^{eg1}/*Tm1*^{eg9} mutant ovaries.

Appendix Figure S2, related to Fig 6:

(A) Khc accumulates around the nurse cells independently of the size of the nucleus. Fitted lines are shown, grey shaded areas represent 95% confidence intervals. R-values of Pearson’s correlation are in the range of -027 to 0.28, however, none of the fitted slopes were found to be significantly different from zero ($p>0.05$).

(B) Mean distribution profile of Dynein intermediate chain (Dic, black) and Khc (grey) around the nurse cell NE in wild-type egg-chambers. Lines indicate mean and 95% confidence intervals.

(C-E) Localization of Egalitarian (Egl, C) dynactin complex member Dynamitin (dmn, D) and the dynein holoenzyme component dynein intermediate chain (Dic, E) in stage 6-8 wild-type egg-chambers.

(F-H) Khc distribution in egg-chambers expressing *oskar Δi(1,2,3)* (F), *oskar 3'UTR* (G) or *Khc* RNAi (H) in the female germ-line.

Scale bars are 50 μm.

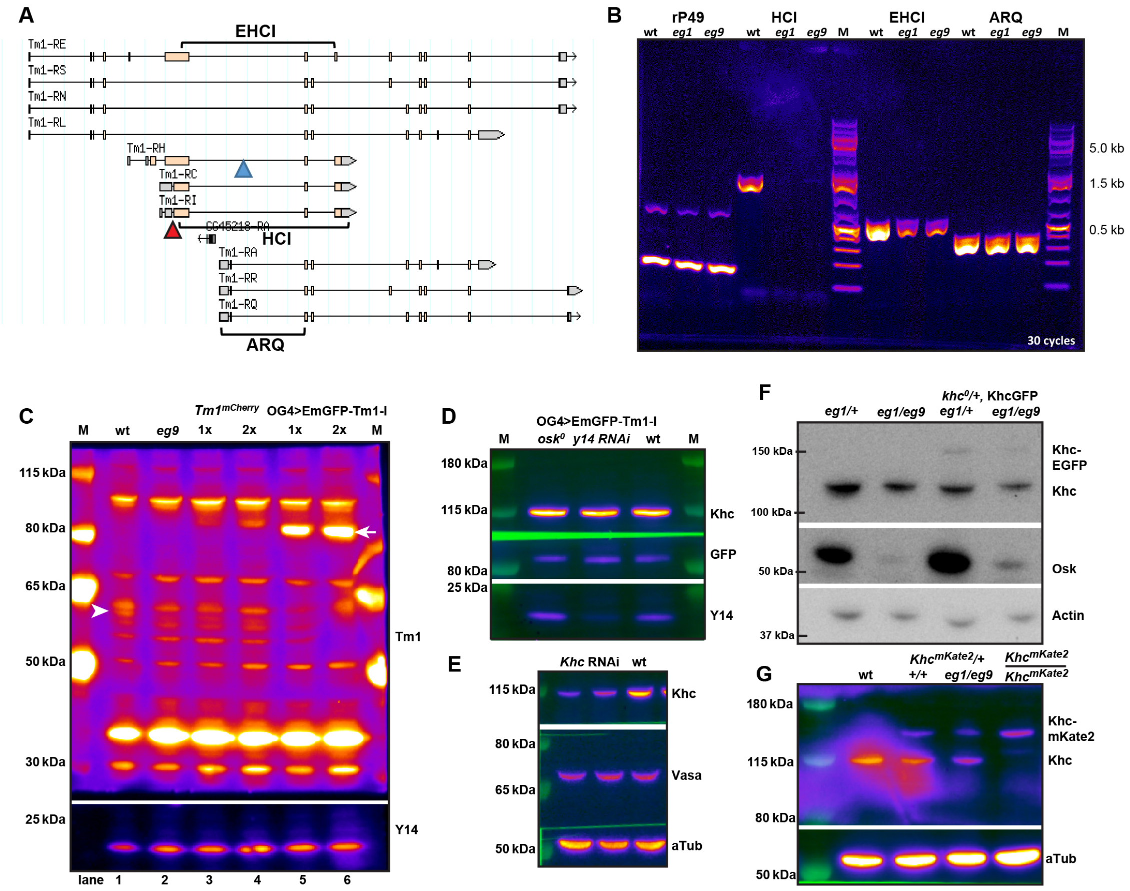

Appendix Figure S1

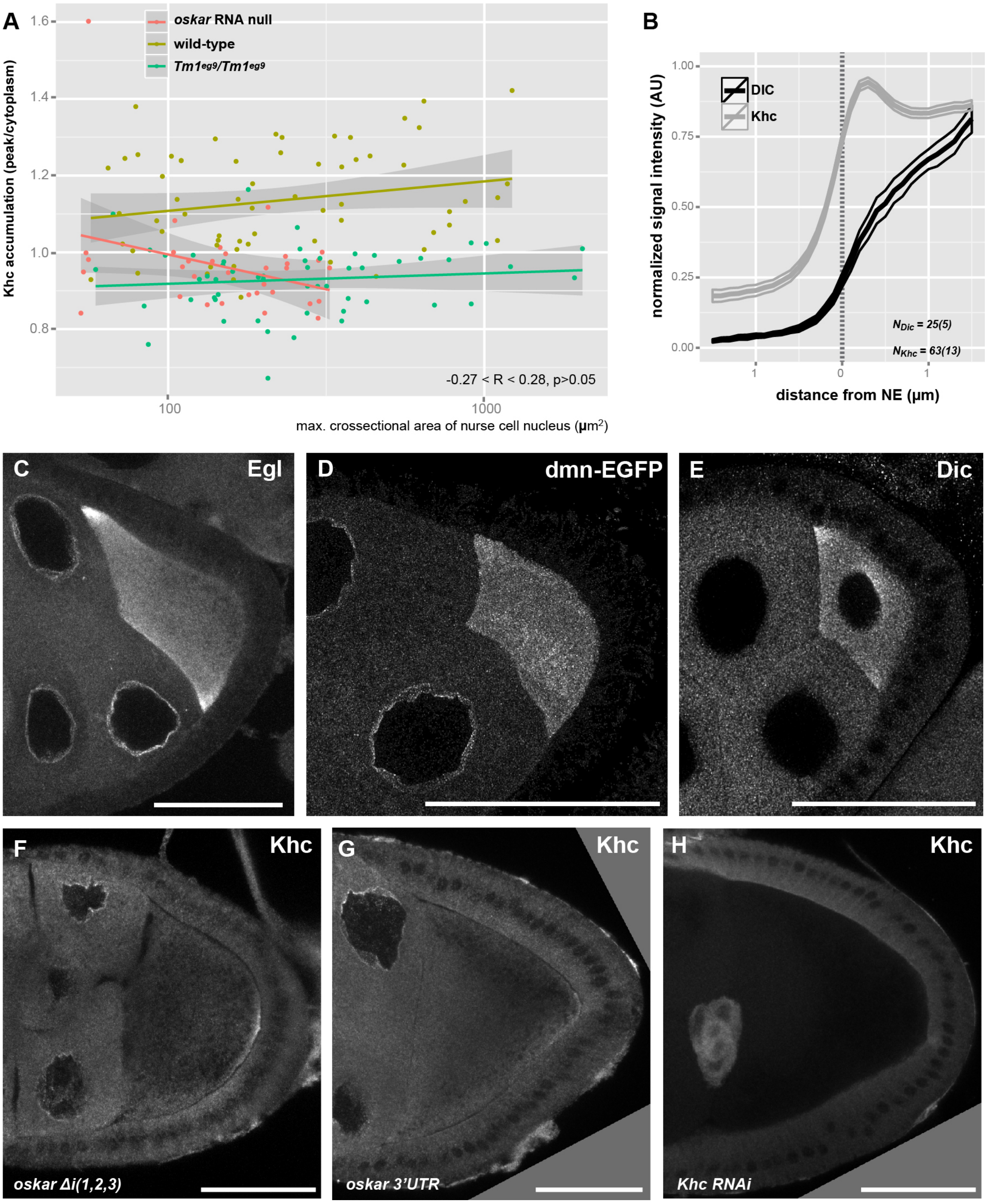

Appendix Figure S2
