# Supplementary material for: An RNA‐binding atypical tropomyosin recruits kinesin‐1 dynamically to oskar mRNPs
Source: EMBO J. 2016 Dec 27;36(3):319–33. doi: 10.15252/embj.201696038 (PMC5286366; doi:10.15252/embj.201696038)
Supplement: Supplementary file 2 — Expanded View Figures PDF [file EMBJ-36-319-s002.pdf]

Expanded View Figures

Figure EV1. *Ex vivo* RNP motility and colocalization assays.

A Schematic of *ex vivo* ooplasmic preparation imaged with TIRF microscopy.

B Distribution of *oskMS2*-GFP mRNP runs towards plus (white) and minus ends (grey). Black boxes indicate the fraction of RNP runs to which no polarity could be assigned (n.d.). Numbers within the bars indicate the number of runs.

C, D Speed (C) and displacement (D) of *oskMS2*-GFP mRNP runs towards plus and minus ends of MTs. Bars indicate wild-type (green), $Tm1^{eg1}/Tm1^{eg9}$ (orange), Khc-null (white) and $Tm1^{eg1}/Tm1^{eg9}$ + Khc_{401} ooplasms (light blue), from left to right. The parameters are statistically not different from those determined in the wild-type control ($P > 0.05$), with exception of the plus-end-directed transport length observed in $Tm1^{eg1}/Tm1^{eg9}$ extracts ($^*P = 1.4 \times 10^{-4}$, pairwise Mann–Whitney *U*-test). Runs of undetermined polarity were excluded from testing. Number of runs analysed is indicated in panel (B). The bottom and the top of the box represent the first and third quartiles, the thick horizontal lines indicate the data median. Whiskers show the data range excluding outliers, which are represented by dots.

E, F Observed (points) and estimated random (coloured ribbons) colocalization of *oskMS2-mCherry* mRNPs with different GFP fusion proteins (E) in different ooplasms (F) as a function of colocalization window size. MCP indicates MCP-EGFP which, like MCP-mCherry, can bind to MS2 loops. Numbers indicate the number of particle clusters (160 mRNPs in each) and the number of preparations (in brackets) analysed.

G, H Difference between the observed and estimated random colocalization values. Horizontal black line above the graphs indicates the clipping point and regime when random colocalization dominates the difference values (H). For comparison of colocalization levels, the data points lying midway between zero distance and the onset of the random dominated regime (clipping point) were selected (at 200 nm, indicated by arrowheads). *0* indicates that colocalization values observed in $Tm1^{eg1}/Tm1^{eg9}$-mutant ooplasms using 100-nm colocalization window are not different from zero ($P > 0.05$, one-sample *t*-test).

Data information: (E–H) Error bars represent 95% confidence intervals.

© 2016 European Molecular Biology Laboratory

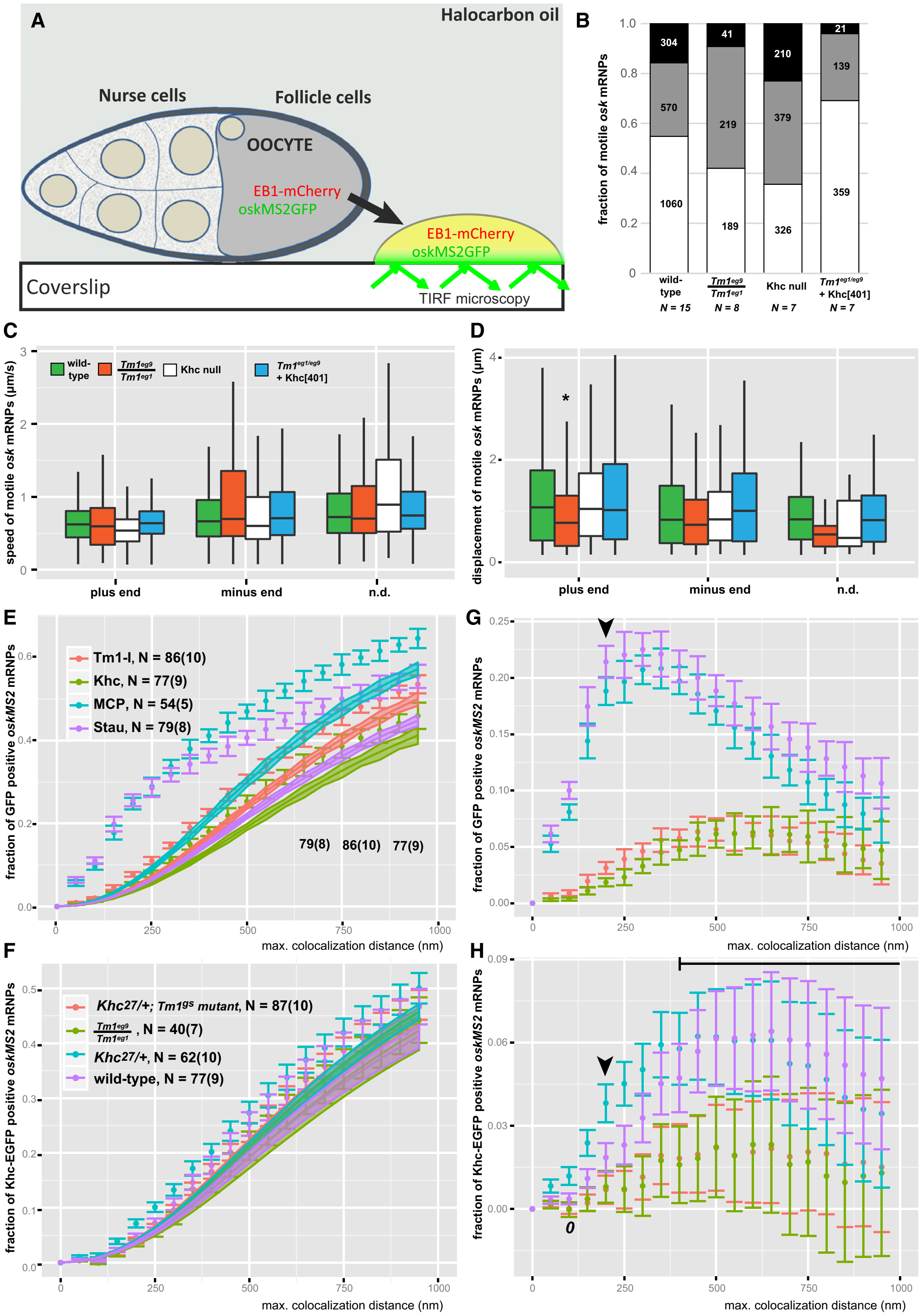
A
Halocarbon oil
Nurse cells
Follicle cells
OOCYTE
EB1-mCherry
oskMS2GFP
Coverslip
TIRF microscopy
B
fraction of motile osk mRNPs
wild-type N = 15
Khc null N = 7
Tm1eg1/eg9 + Khc[401] N = 7
C
speed of motile osk mRNPs (µm/s)
Khc null
plus end
minus end
n.d.
D
displacement of motile osk mRNPs (µm)
E
Tm1-I, N = 86(10)
Khc, N = 77(9)
MCP, N = 54(5)
Stau, N = 79(8)
fraction of GFP positive oskMS2 mRNPs
max. colocalization distance (nm)
F
N = 40(7)
Khc27/+, N = 62(10)
wild-type, N = 77(9)
fraction of Khc-EGFP positive oskMS2 mRNPs
G
H

Figure EV1.

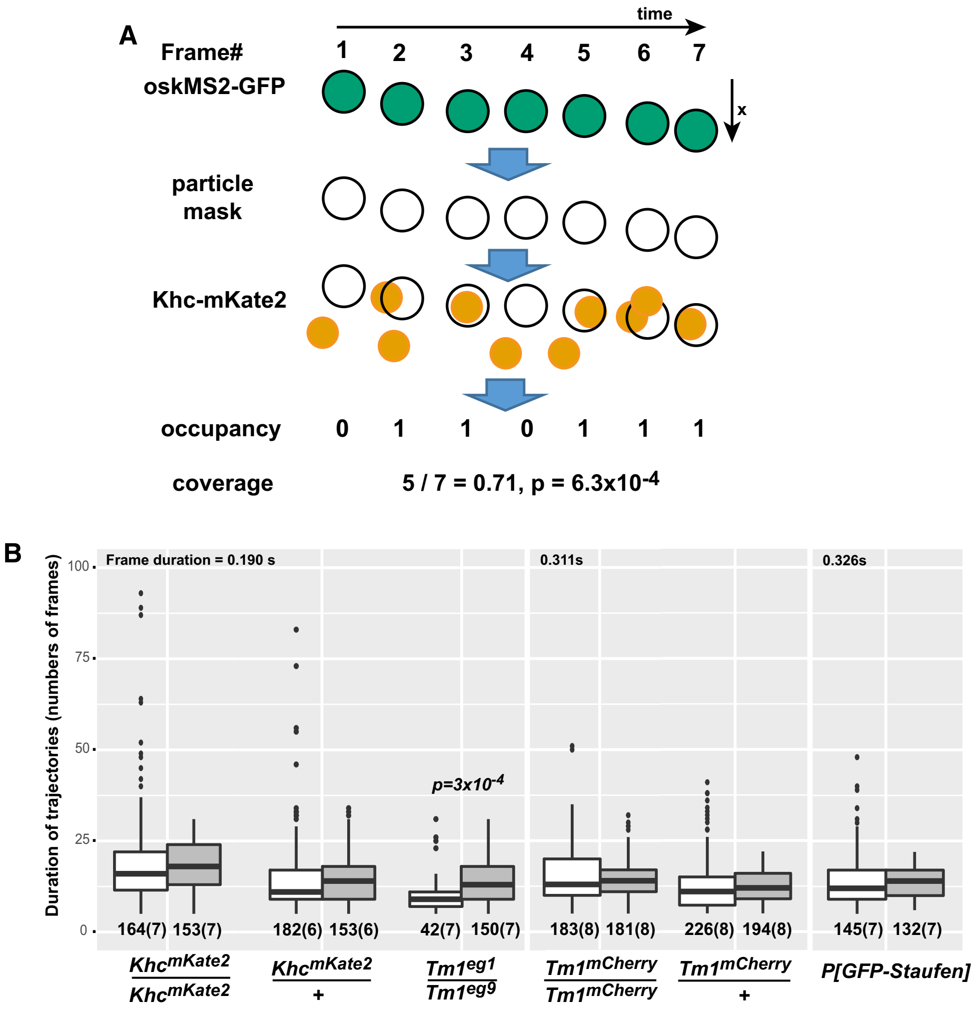

Figure EV2. ***Ex vivo*** **temporal colocalization assay.**

A Schematics of temporal colocalization. *oskarMS2* mRNPs were tracked in a manually assisted way. The mRNP particle outlines during a trajectory were used as a mask to measure the fluorescence of the fluorescently tagged protein molecules (e.g. Khc-mKate2). The measured mean signal intensities of the assayed protein were compared to a threshold during every frame of the trajectory to determine colocalization of the protein of interest and the tracked mRNP (occupancy). These threshold values were determined as the lower 10th percentile of the mean signal intensity distribution of all observed assayed protein "particles" (not exclusively those under the mask) in heterozygous extracts. None of the threshold values of a given protein molecule determined in individual extracts differed by more than 10% of their mean. The mean threshold value was used for a given protein both for hetero- and homozygous extracts. The sum of the occupancies divided by the total number of frames in the trajectory defined a single coverage value, which is plotted in Fig 3C–E. The probability of random occurrence of a coverage sequence was determined by calculating the corresponding frequency of a binominal distribution (*Pr*). As a "success probability in each trial" (*P*) measure, we used the estimated random colocalization values of each protein observed at ~250 nm max distance, since the particle masks had an average diameter of 250 nm: for Khc-mKate2, mCherry-Tm1 and GFP-Staufen, these *P*-values were 0.132, 0.077 and 0.08, respectively. We scored a colocalization event as highly confident when the calculated *Pr* value was smaller than 0.01 and the coverage was ≥ 0.5. The fraction of these events is indicated above the boxplots in Fig 3C–E.

B Duration of motile (white) and non-motile (grey) *oskar* mRNP trajectories. To avoid bias during the determination of high-confidence association, a population of non-motile RNPs was selected whose duration did not differ significantly ($P > 0.05$, pairwise Mann–Whitney *U*-test) from that of the corresponding motile RNP population. The $Tm1^{eg1}/Tm1^{eg9}$ data were an exception, as in this case we observed a significant difference between the duration of motile and non-motile trajectories; however, there was no significant difference between the Khc coverage of the two motility categories ($P = 0.023$, Fig 3C). Frame duration (temporal resolution) is indicated above the boxplots. Numbers below the boxplots indicate the number of trajectories and the number of ooplasms (in brackets) analysed. The bottom and the top of the box represent the first and third quartiles, the thick horizontal lines indicate the data median. Whiskers show the data range excluding outliers, which are represented by dots.

 © 2016 European Molecular Biology Laboratory

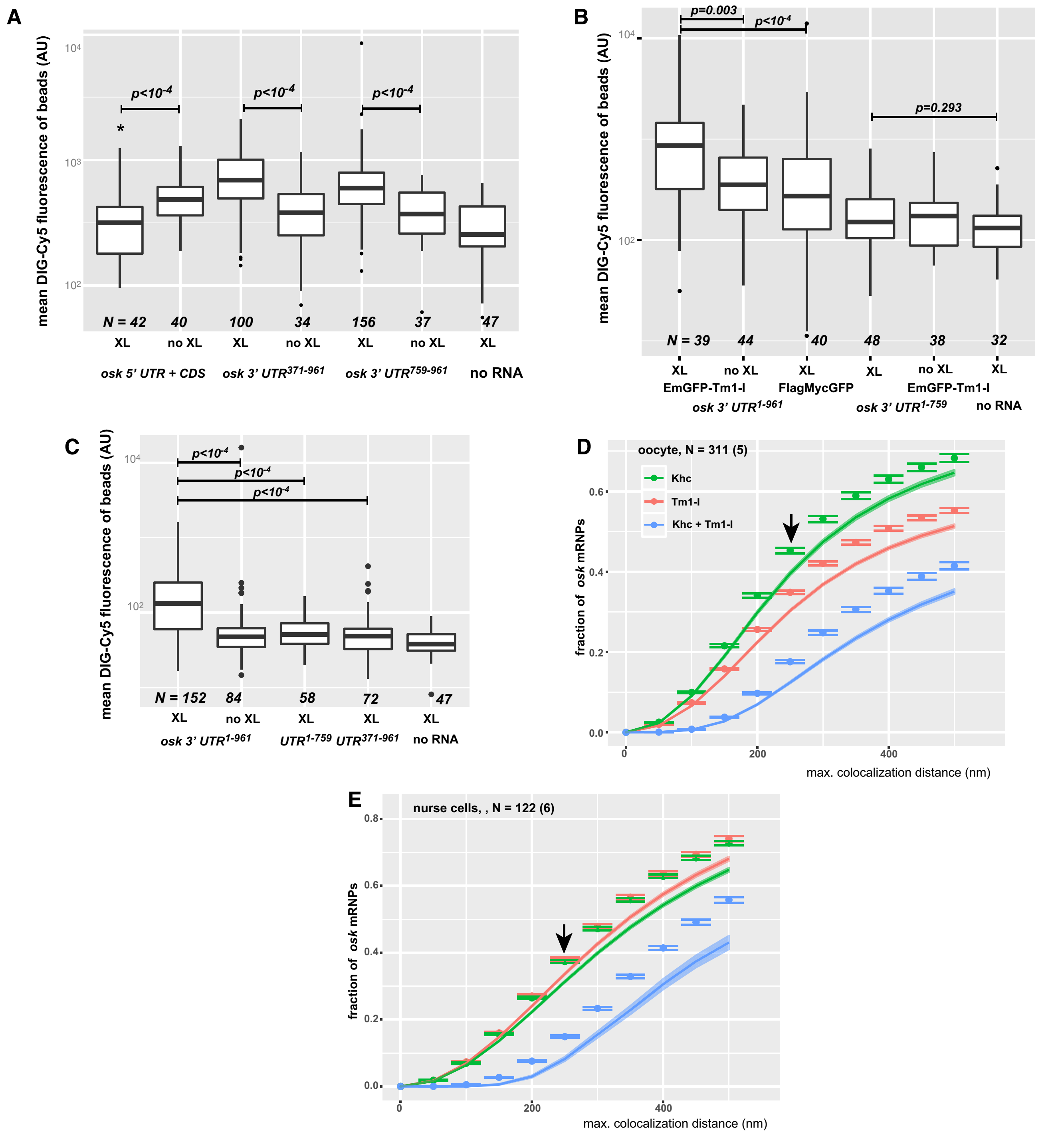

Figure EV3. RNA/RNP binding property of Tm1-I/C *in vitro* and *in situ*.

A–C Mean DIG-Cy5 fluorescence of GFP-Trap_M beads in the presence of the indicated RNA fragments with and without UV cross-linking. In the case of the FlagMycGFP negative control (B) and the truncated, non-binding *oskar* 3′UTR fragments (C), non-cross-linking conditions were not tested. *P*-values of pairwise Mann–Whitney *U*-tests are indicated. Numbers indicate the number of beads analysed. The bottom and the top of the box represent the first and third quartiles, the thick horizontal lines indicate the data median. Whiskers show the data range excluding outliers, which are represented by dots.

D, E Observed (points) and estimated random (coloured ribbons) colocalization of *oskar* mRNPs with Khc-mKate2 (green) or EmGFP-Tm1-I (red) or with both fusion proteins (blue) in wild-type oocytes (C) and nurse cells (D). Arrows indicate the maximal colocalization distance (250 nm) chosen to analyse *oskar* mRNP composition in Fig 5F–I. The expected value of independent interaction was calculated by taking the difference of the products of observed individual colocalizations (Khc or Tm1-I) and of expected individual colocalizations. Numbers indicate the number of particle clusters (100 *oskar* mRNPs in each) and the number of egg-chambers (in brackets) analysed. Error bars represent 95% confidence intervals.

© 2016 European Molecular Biology Laboratory

Figure EV4. Composition of *oskar* mRNPs *in situ*.

A–A″ Confocal image of a wild-type egg-chamber expressing GFP-Mago (green, A′) and mKate2-Tm1-I (magenta, A″). *oskar* mRNA labelled with osk1-5 FIT probes is in yellow.

B Fraction of *oskar* mRNPs colocalizing with GFP-Mago (green), mKate2-Tm1-I (orange), or both of these proteins (light grey) in the oocyte or in the nurse cells (max. colocalization distance is 250 nm). Horizontal dashed lines indicate the expected value of observing both protein in an *oskar* mRNP if the interactions are independent (see legend to Fig EV3D and E). Significance of the observed colocalization values versus the expected values is shown. In the nurse cells, GFP-Mago and mKate2-Tm1-I appear to be independent ($P > \alpha = 0.001$, one-sample *t*-test), whereas in the oocyte the presence of the two proteins on *oskar* mRNPs appears to anti-correlate.

C, C′ Example confocal (C) and gated STED image (C′) of EmGFP-Tm1-I-expressing nurse cells. EmGFP-Tm1-I is in magenta, and *oskar* mRNA is in green.

D Difference between observed and estimated random colocalization of *oskar* mRNPs with *oskMS2*(*10x*)-MCP-GFP (black), EmGFP-Tm1-I (orange), Khc-EGFP (blue) and Ketel-GFP (importin-β, red) in STED images. At 100 nm maximal distance, *oskar* mRNPs barely colocalize with the negative control Ketel-GFP ($P = 0.012$, one-sample *t*-test versus zero). All other colocalization values are significantly different from that observed for Ketel-GFP ($P < 0.005$, two-sample *t*-test).

E Fraction of Khc-EGFP-positive *oskar* RNPs in the indicated nurse cells. *P*-values of two-sample *t*-tests against wild type are indicated.

F, G Mean density of detected *oskar* mRNA (dark grey), GFP-Mago (green), mKate2-Tm1-I (orange) and Khc-mKate2 (blue) in the nurse cell nuclei, cytoplasm and the ooplasm (F) and in the nurse cells under the conditions tested (G). All values are significantly different from zero ($P < 0.05$, one-sample *t*-test). Khc is considered to be a ubiquitous cytoplasmic protein, and therefore, we took its nuclear density as an estimate of the errors of the analysis (e.g. false positive recognition and uncertainties during definition of the nucleo-cytoplasmic border). All other nuclear density values are significantly different from that of Khc-mKate2 (F, $P < 0.05$, two-sample *t*-test). None of the Khc-mKate2 and *oskar* mRNA density values are significantly different in the appropriate pairwise comparisons (G, $P > 0.05$). Numbers in the bars show the number of samples analysed.

H Fraction of nuclear *oskar* mRNPs colocalizing with mKate2-Tm1-I (orange), GFP-Mago (green) or Khc-mKate2 (blue). The observed Tm1-I/C- or Khc-positive mRNPs are not different from zero (*P*-values of one-sample *t*-tests are indicated, $\alpha = 0.01$).

I Fraction of Khc-mKate2-positive *oskar* mRNPs containing the full-length mRNA (wild type) or only the *oskar* 3′UTR in the nurse cells. *oskar* mRNA was detected using a set of 15 singly labelled probes against the *oskar* 3′UTR.

J Mean density of the detected Khc-mKate2 (blue) and mRNPs labelled by the *oskar* 3′UTR probe set (grey) in the nurse cells ($N = 5$ each).

Data information: Numbers in the bars indicate the number of particle clusters (number of egg-chambers) analysed. Error bars represent 95% confidence intervals. Scale bars represent 50 μm (A″) and 1 μm (C′).

© 2016 European Molecular Biology Laboratory

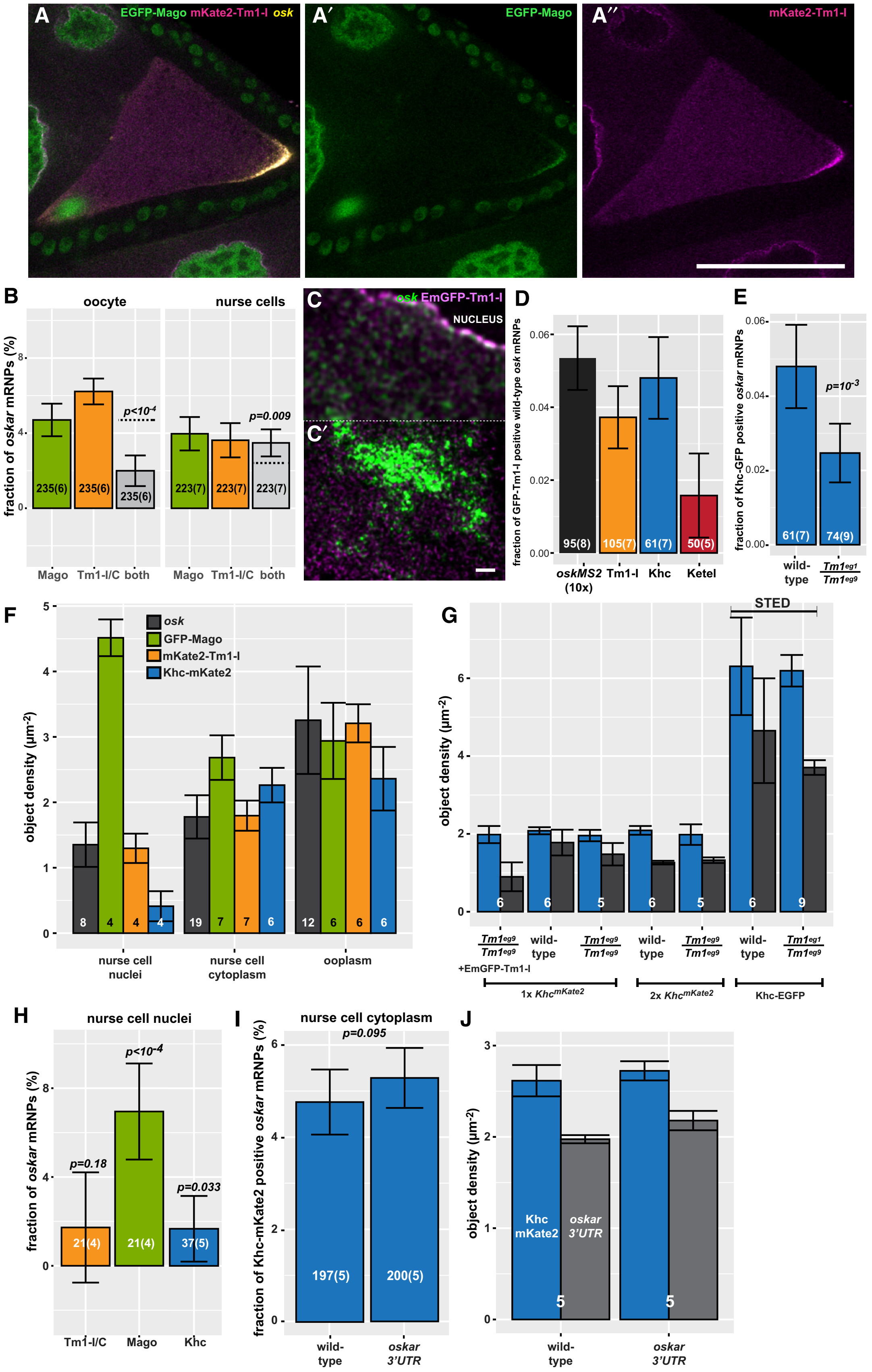

A
EGFP-Mago mKate2-Tm1-I osk
A′
EGFP-Mago
A″
mKate2-Tm1-I
B
oocyte
nurse cells
fraction of oskar mRNPs (%)
p<10-4
p=0.009
Mago Tm1-I/C both Mago Tm1-I/C both
C
osk EmGFP-Tm1-I
NUCLEUS
C′
D
fraction of GFP-Tm1-I positive wild-type osk mRNPs
oskMS2 (10x) Tm1-I Khc Ketel
E
fraction of Khc-GFP positive oskar mRNPs
p=10-3
wild-type
F
object density (µm-2)
osk
GFP-Mago
mKate2-Tm1-I
Khc-mKate2
nurse cell nuclei
nurse cell cytoplasm
ooplasm
G
STED
object density (µm-2)
+EmGFP-Tm1-I
1x Khc mKate2
2x Khc mKate2
Khc-EGFP
H
nurse cell nuclei
fraction of oskar mRNPs (%)
p=0.18
p<10-4
p=0.033
Tm1-I/C Mago Khc
I
nurse cell cytoplasm
p=0.095
fraction of Khc-mKate2 positive oskar mRNPs (%)
wild-type
oskar 3′UTR
J
object density (µm-2)
Khc mKate2
oskar 3′UTR
wild-type
oskar 3′UTR

Figure EV4.

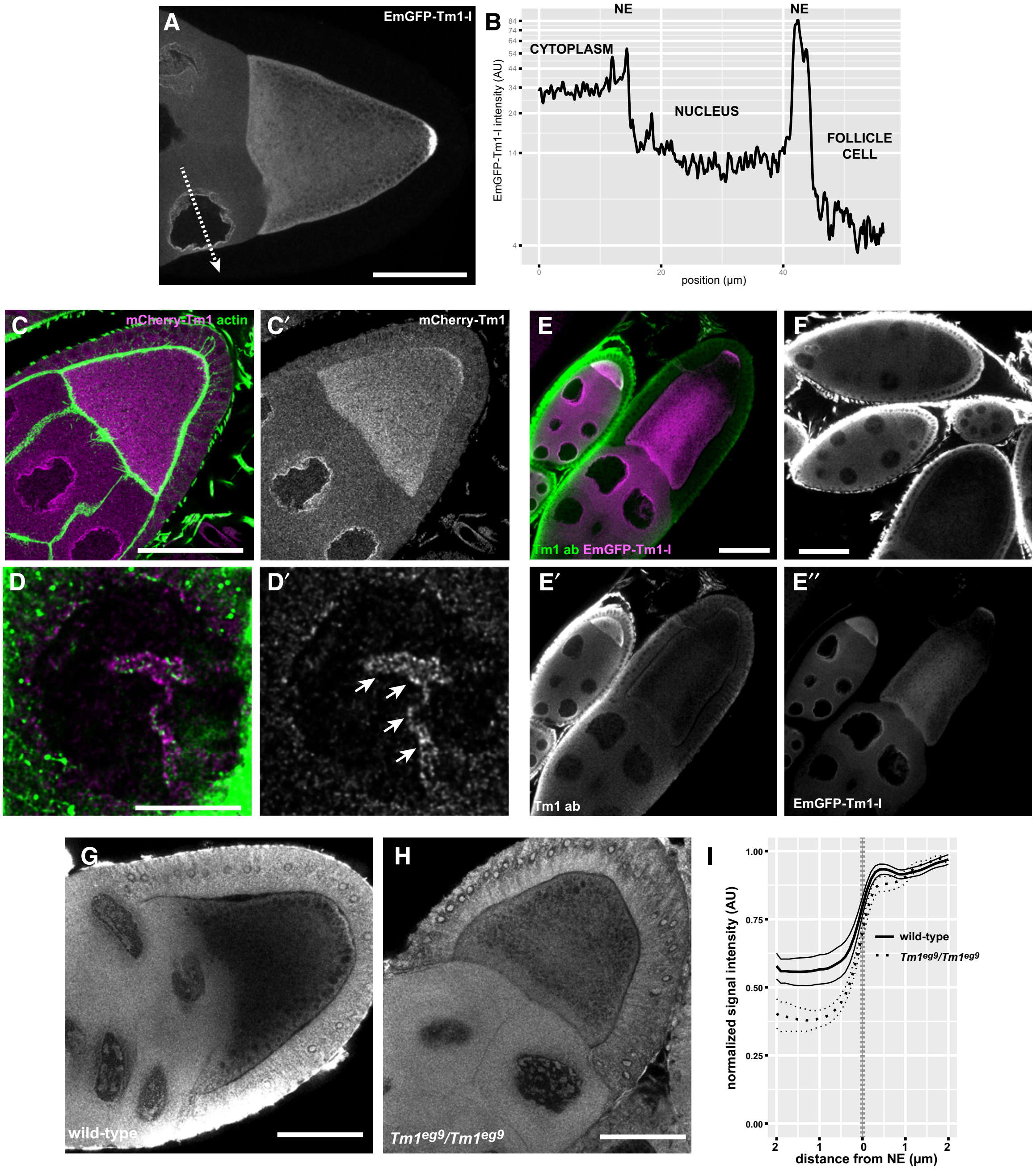

Figure EV5. Localization of Tm1-I/C.

A Localization of EmGFP-Tm1-I expressed within the female germline.

B Intensity profile of EmGFP-Tm1-I along the dashed arrow in (A). Cellular compartments are indicated above the curve. The non-EmGFP-Tm1-I-expressing follicle cells serve as a background reference.

C–D′ Localization of mCherry-Tm1 (magenta) relative to actin (green) within egg-chambers. (D and D′) High-magnification imaging of nurse cells reveals dim actin fibres surrounding the nuclei. However, mCherry-Tm1 puncta do not appear to co-align with these fibres. An infolding of the NE (D′, arrows) appears as an enrichment of mCherry-Tm1 and actin inside the nucleus.

E, F Immunostaining of EmGFP-Tm1-I-expressing (E–E″) and wild-type (F) PFA-fixed egg-chambers with pan-Tm1 antibody. The Tm1 antibody staining (green) shows a signal distribution similar to that of EmGFP-Tm1-I (magenta) within oocytes; however, Tm1-I localization around the nurse cell NE is barely recognizable in PFA-fixed mid-oogenetic egg-chambers (E′ and F).

G–I In heat-fixed specimen, on the other hand, we could detect a faint perinuclear accumulation of the pan-Tm1 antibody signal in wild-type (G) but not in *Tm1*^{gs}-mutant (H) stage 8–10 nurse cells. The subtle accumulation we could quantify (I: N_{wt} = 23, N_{Tm1gs} = 18). Lines indicate mean and 95% confidence intervals.

Data information: Scale bars represent 50 µm (A, C, E–H) and 10 µm (D).

 © 2016 European Molecular Biology Laboratory
