# Supplementary material for: An RNA‐binding atypical tropomyosin recruits kinesin‐1 dynamically to oskar mRNPs
Source: EMBO J. 2016 Dec 27;36(3):319–33. doi: 10.15252/embj.201696038 (PMC5286366; doi:10.15252/embj.201696038)
Supplement: Supplementary file 3 — Video EV1 [file EMBJ-36-319-s003.zip › EMBOJ_96038_VideoEV1/Video_EV1.docx]

**Video EV1, related to Figure 1:** *oskar* mRNP (green) motility in wild-type *ex vivo* ooplasmic preparation. 20 frame running average of EB1-mCherry signal is shown in magenta to highlight dynamic MTs. Scale bar is 5 µm.
