# Supplementary material for: An RNA‐binding atypical tropomyosin recruits kinesin‐1 dynamically to oskar mRNPs
Source: EMBO J. 2016 Dec 27;36(3):319–33. doi: 10.15252/embj.201696038 (PMC5286366; doi:10.15252/embj.201696038)
Supplement: Supplementary file 4 — Video EV2 [file EMBJ-36-319-s004.zip › EMBOJ_96038_VideoEV2/Video_EV2.docx]

**Video EV2, related to Figure 2:** Colocalization of Khc-EGFP (green) with *oskMS2*-mCherry mRNPs (magenta) in wild-type *ex vivo* ooplasmic preparations. White and yellow arrowheads indicate motile and non-motile oskar mRNPs co-localizing stably with the fluorescently tagged protein of interest, respectively. Scale bar is 5 µm.
