# Supplementary figures and images for: An RNA‐binding atypical tropomyosin recruits kinesin‐1 dynamically to oskar mRNPs
Source: EMBO J. 2016 Dec 27;36(3):319–33. doi: 10.15252/embj.201696038 (PMC5286366; doi:10.15252/embj.201696038)

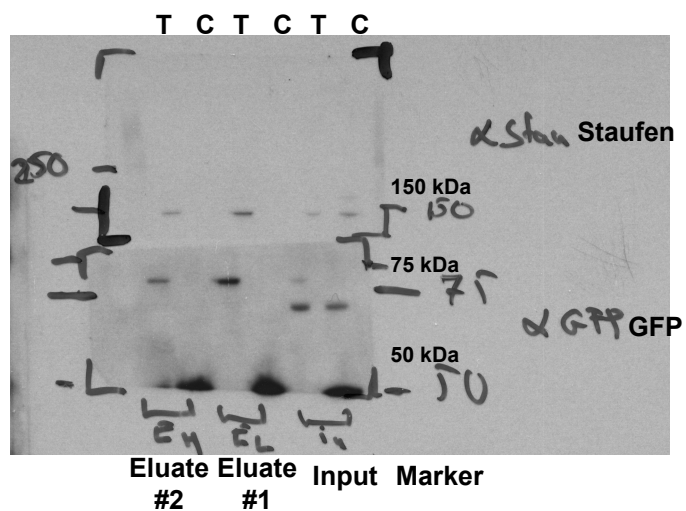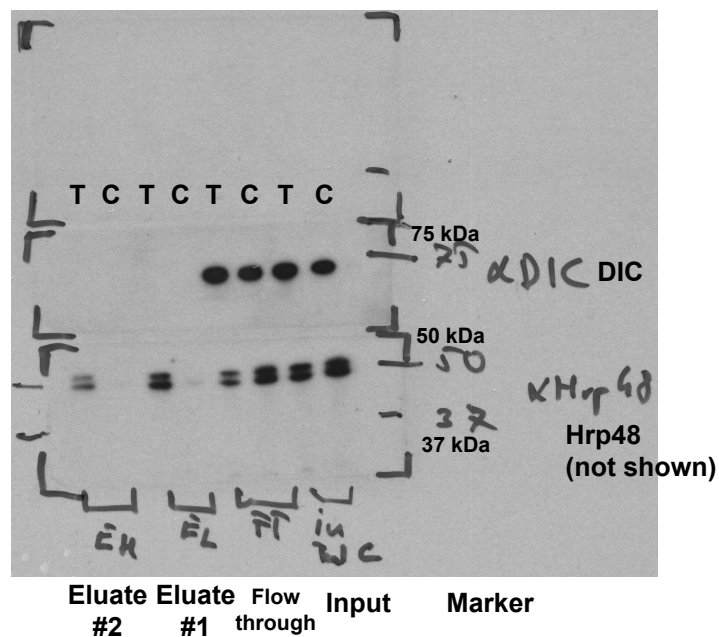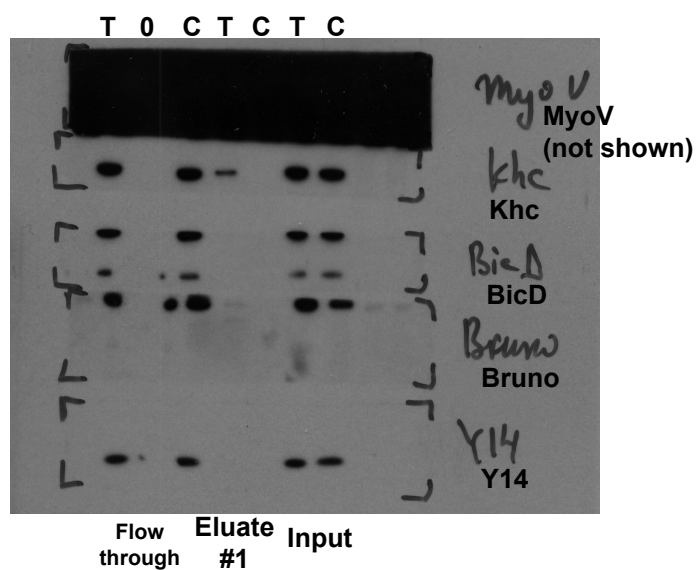

Source data of Figure 4A

Supplement: Supplementary file 8 — Source Data for Figure 4A [file EMBJ-36-319-s007.pdf]
